# Supplementary material for: Evaluating equality in prescribing Novel Oral Anticoagulants (NOACs) in England: The protocol of a Bayesian small area analysis
Source: PLoS One. 2021 Feb 4;16(2):e0246253. doi: 10.1371/journal.pone.0246253 (PMC7861433; doi:10.1371/journal.pone.0246253)
Supplement: Attachment — Submitted filename: Response to Reviewers.docx [file pone.0246253.s001.docx]

**Manuscript PONE-D-20-10082**

**Respond to Reviewers**

Dear Dr Tizzoni,

Thank you for giving us the opportunity to submit a revised draft of the manuscript “Evaluating Equality in Prescribing Novel Oral Anticoagulants (NOACs) in England: Protocol of a Bayesian Small Area Analysis” for publication in the PLOS ONE journal. We appreciate the time and effort that you and reviewers dedicated to providing feedback on our manuscript and are grateful for the insightful comments on and valuable improvements to our paper. We have incorporated all of the suggestions made by the reviewers. Those changes are highlighted in the manuscript. Please see below, in blue, for a point by point response to reviewer’s comments and concerns.

Please note that all page numbers refer to the revised manuscript file “with track changes”.

**Review Comments to the Author**

**Reviewer #1:** The paper describes a protocol for a study that will estimate the equality in prescribing individual NOACs with high spatial resolution in Clinical Commissioning Groups (CCGs) across England from 2013 to 2019, using Bayesian spatio-temporal modelling.

The introduction is focussed on the positive aspects of NOACs and does not refer to the negative or adverse effects associated with these medicines. A balanced review would be useful.

Author response: Thanks for pointing this out. We have added negative aspects of NOACs. The revised text demonstrates on page 4, paragraph 2 under background section.

Placement of references should be at the end of a statement/sentence not before e.g. ‘These NOACS… for (5-8) 1)…’ it is confusing to have the references and then numbering of points.

Author response: As suggested by the reviewer, we have changed the references location from the middle of sentences to the end.

Page 4 – ‘epidemic of atrial fibrillation’ is used but ‘epidemic’ is probably not the right word to use here.

Author response: Thanks for pointing this out. We have changed the “epidemic of atrial fibrillation” to “prevalence of atrial fibrillation” on page 5.

Aims and questions section appears very long. The first sentence requires re-wording – it may not be required since it is covered already.

Author response: We think this is an excellent suggestion. We have summarised and done rewording for the aims and questions on page 5 and 6.

Methods – is the prescribing data linked to diagnosis? Will other indications other than Afib be considered in the analysis?

Author response: The prescribing data does not contain information regarding diagnosis. An explanation has been added under data sources subtitle on page 9, paragraph 1.

A reference to the ‘non-optimal’ prescribing is required.

Author response: We appreciate reviewer’s suggestion. Reference has been added on page 6 under Rationale for analysing NOACs section (Reference 18).

How is clustering within areas accounted for?

Author response: Clustering effects within the CCGs are part of the model’s error term. Details have been added and explained in the small area estimation model subsection on page 11, paragraph 2.

2019 is used as a reference year, but the data are available april 2013-april 2019, so is it the first 4 months of 2019 is reference or the year May2018-April 2019? Not clear.

Author response: Thanks for pointing this out. We have modified these sentences for more clarification on page 8 under the reference year and data sources subsections.

How far forward will be considered for predictions?

Author response: We aimed to predict for the future estimates. However, due to COVID situation and data quality for year 2020, we have updated the reference year section on page 8.

Could age/gender specific DDDs be considered, as large differences in age and gender specific prescribing?

Author response: DDDs are similar across age and gender categories. More explanation about this question has been updated on page 4 in Background section and page 8 under Standardising different dosages and primary variable of interest section.

Analysis and modelling section – more explanation of the Bayesian models would be included and why they are used over non-Bayesian approaches? Also, a brief intro to Hierarchical modelling – not all readers may be familiar.

Author response: As per your suggestion, more explanation about Bayesian and non-Bayesian approaches, plus hierarchical modelling has been updated in section analysis and modelling on page 9 and 10, paragraphs 1 and 2.

Statement ‘BYM introduced by (30)….’ Please give authors name etc and not a reference. Also how does BYM address the issue with inter-dependency? Some explanation would be helpful.

Author response: Thanks for pointing this out. We have modified this reference format to the name of the author. Also, more explanation about BYM model issues and the improvements on this model has been updated in section analysis and modelling on page 10, paragraphs 3.

How was the temporal term defined? Monthly, yearly??

Author response: Thanks for pointing this out. We mentioned “yearly” time scale on page 12 under the small area estimation section.

SAE model – provide references or explain the Bayesian Hierarchical framework. This section could be much clearer – particularly when explaining ‘spatial variation due to different…. By this combined parameter. This was not clear.

Author response: We have added more explanations on analysis and modelling, and also in small area estimation sections. We have clarified the hierarchical models and its application on the SAE model. The majority of modifications in the manuscript are related to this comment. Therefore, we have updated several sentences on pages 11 and 12.

‘NOVAs’ instead of ‘NOCAs’ needs correcting.

Author response: Thanks for pointing this out. The word has been corrected on page 11, paragraph 3.

Explain why using log normal used?

Author response: This is because in the Poisson model we used, a general model formulation assumes that SAE components have the association with log of DDDs rate. We have modified the formulation of the model and clarified the definitions. More explanation has been added on page 11 under the section small area estimation model.

Formula is used without explanation of the meaning of the parameters, It is assumed the reader is aware of these.

Author response: We have added more explanations and definition for parameters used in the SAE model on pages 11 and 12.

For the explanation of Rstan ‘Stan used no … from posterior’ requires further referencing. Why does NUTs sampler make ‘appropriate estimate’ over others – the justification is not evident.

Author response: We have explained NUTS and stan package; and also added references for Rstan package and stan development team on page 12.

Assumptions – how will these assumptions be tested? Should sensitivity analysis be applied here?

Author response: The assumptions are being made as they have implications in the interpretations of the model’s output. If any of the assumptions seems to be true, the main way of checking its effects on the model’s output will be through empirical defining counterfactual scenarios *a posteriori* and rerunning the model.

We have added this sentence on page 13 under the assumption section to make it clear.

Validation – presented in the ‘past’ tense which reads as if this work/analysis has been done already, even though this is a protocol for the analysis. Please correct.

Author response: Thanks for pointing this out. All the tense mistakes in the validation section have been fixed on page 13.

Ethical considerations – similarly using ‘past’ tense. Is this appropriate?

Author response: Thanks for pointing this out. All the tense mistakes in the ethical consideration section have been fixed on page 13.

Discussion – there is some repetition with what is included in the introduction - is this required in a protocol?

Author response: Thanks for pointing this out. We have fixed these repetitive sentences in the discussion on page 14.

There is no mention of whether the data and/or models will be made available through open source platforms. This is required.

Author response: We appreciate this valuable comment. We have added a subsection entitled open data sharing on page 14.

The level of English could be improved e.g. pg 2 discussion last sentence ‘can be monitor for a long time’ rather than ‘monitored’. A thorough proof-read of the paper is required.

Author response: Thanks for your helpful suggestion. We have thoroughly proof-read the paper.
