# Supplementary material for: Evaluating equality in prescribing Novel Oral Anticoagulants (NOACs) in England: The protocol of a Bayesian small area analysis
Source: PLoS One. 2021 Feb 4;16(2):e0246253. doi: 10.1371/journal.pone.0246253 (PMC7861433; doi:10.1371/journal.pone.0246253)
Supplement: Attachment — Submitted filename: Response to Reviewers.docx [file pone.0246253.s002.docx]

**Manuscript PONE-D-20-10082**

**Respond to Reviewers**

Dear Dr Tizzoni,

Thank you for giving us the opportunity to submit a revised draft of the manuscript “Evaluating Equality in Prescribing Novel Oral Anticoagulants (NOACs) in England: Protocol of a Bayesian Small Area Analysis” for publication in the PLOS ONE journal. We appreciate the time and effort that you and reviewers dedicated to providing feedback on our manuscript and are grateful for the insightful comments on and valuable improvements to our paper. We have improved the language using a professional English language editing system. We have incorporated all of the suggestions made by the reviewers. Those changes are highlighted in the manuscript. Please see below, in blue, for a point by point response to reviewer’s comments and concerns.

Please note that all page numbers refer to the revised manuscript file “with track changes”.

**Review Comments to the Author**

**Reviewer #1:** Most of the previous points have been addressed - however, there are two further minor corrections required.

1. Original Question in first review: Could age/gender specific DDDs be considered, as large differences in age and gender specific prescribing?

The response and change to the manuscript text is not what was requested. I understand that the data are not available by age or gender, and therefore, the following sentence in methods can be removed as it adds confusion rather than clarity.

‘DDD is not age- or gender-specific, and therefore, it can suitably be used in our modelling which is on population-level, aggregate prescription data without gender or age specifications.’

Author response: Thanks for pointing this out. We removed this sentence as you mentioned.

2. Thorough Proof-read: Despite a request for a thorough proof-read for English, some errors still need to be addressed. A list of examples is provided, but not exhaustive:

In the methods
‘Health economical ‘– change to health economics
‘In compliance with the copy rights,’ – change to ‘in compliance with copyright’
‘Due to insufficient sample size prescribed at the small levels, direct estimators are unreliable to provide adequate..’ - please rephrase ‘at the small levels’ so it is clear what this means?

‘In Bayesian approach’ – should be ‘In the Bayesian approach..’
‘These distributions control the parameters limits that they can vary in the model.’ – rephrase
‘Since the non-spatial random effect is included to capture for independent region-specific variation, most or all of the variation can be addressed.’ – rephrase e.g. ‘Since the non-spatial random effect is included to enable the capture of the independent region-specific variation,….’

‘precision parameter for combined component and a mixing parameter..’ – change to ‘precision parameter for the combined component and a mixing parameter’

‘The effects of clustering within the CCGs (….) will show itself in the remainder error terms of the model.’ – change to ‘the effect of clustering with the CCGs () will be present in the remaining error terms…’

‘If any of the assumptions seems to be true, the main way … through empirical defining counterfactual..’ – change to ‘ If any of the assumptions appear to be true, the main way … through empirically defining counterfactual’

Author response: Thanks for your helpful suggestions. We have thoroughly proof-read the paper. In addition to all your corrections, we also used a professional English editing system to address all the language mistakes in the manuscript.
